# Prevalence and Determinants of Migraine Among College Students in Ernakulam, Kerala, India: A Cross-Sectional Study

**DOI:** 10.7759/cureus.103352

**Published:** 2026-02-10

**Authors:** Shobhiyaa Panneer, Tiyasha Nandi, Saisri Tanya Kamisetti, Sandra Saji, Shaambhavi Singh, Shantanu Menon, Sarah Thomas, Tatineni Devi Manogna, Sean George, Parvathi Sureshkumar, Shifa Habeeb, Brilly M Rose, Navami S

**Affiliations:** 1 Community Medicine, Amrita Institute of Medical Sciences, Amrita Vishwa Vidyapeetham, Kochi, Ernakulam, IND

**Keywords:** burden, id-migraine, lifestyle factors, migraine, pss-10, screening

## Abstract

Background

Migraine is a common and disabling neurological disorder among young adults that can impair academic performance, social functioning, and the quality of life. Commonly reported triggers include psychological stress, inadequate sleep, and prolonged screen exposure; however, migraine is frequently underrecognized or misclassified as a nonspecific headache. This study aimed to assess the prevalence of probable migraine and examine its association with selected sociodemographic and lifestyle factors among college students in Ernakulam district, Kerala, India.

Methods

A cross-sectional study was conducted among 491 college students over a two-week period. Data were collected using a pretested, semi-structured questionnaire that included the ID-Migraine screening tool to identify probable migraine. Statistical analyses were performed using Jamovi software (retrieved from https://www.jamovi.org). Multivariable logistic regression analysis was carried out to identify factors independently associated with probable migraine, with results expressed as adjusted odds ratios (aORs) and 95% confidence intervals (CIs).

Results

The prevalence of probable migraine was 37.5% (n = 184; 95% CI: 33.2-41.8). The prevalence was higher among women (n = 119, 46.5%) compared to men (n = 65, 27.7%). On multivariable analysis, higher perceived stress, female gender, inadequate sleep (<8 hours/day), and prolonged screen time (>6 hours/day) were independently associated with probable migraine. No statistically significant associations were observed with age, the course of study, accommodation type, physical activity, smoking, alcohol consumption, or substance use.

Conclusion

Probable migraine was highly prevalent among college students in Ernakulam district and was significantly associated with perceived stress, sleep deprivation, female gender, and prolonged screen exposure. These findings underscore the importance of incorporating migraine screening, stress management strategies, the promotion of healthy sleep practices, and guidance on healthy screen use within student health services to improve well-being in this population.

## Introduction

Migraine is a common neurological disorder characterized by recurrent throbbing or pulsatile headaches, frequently accompanied by nausea, vomiting, and increased sensitivity to light and sound [1]. Owing to its disabling nature, migraine substantially disrupts daily activities and adversely affects the overall quality of life. Globally, it is among the most prevalent neurological disorders, disproportionately affecting young adults, and represents a leading contributor to disability-adjusted life years (DALYs) lost among individuals younger than 50 years [2,3]. The World Health Organization (WHO) recognizes migraine as one of the most disabling conditions worldwide, highlighting its significance as a major public health concern [4]. According to the Global Burden of Disease (GBD) 2019 estimates, the number of people living with migraine increased from 721.9 million in 1990 to 1.1 billion in 2019, corresponding to a global prevalence of approximately 14% [5]. The burden is particularly high in low- and middle-income regions of Southeast Asia, where nearly 113 million cases were reported in 2019 [6].

In India, migraine prevalence is markedly higher than the global average, with population-based studies documenting rates of approximately 25% in Karnataka [7], in contrast to the worldwide prevalence of 14% [5]. Despite this considerable burden, migraine continues to be underdiagnosed and inadequately treated, largely due to its heterogeneous symptomatology, overlap with psychiatric comorbidities such as depression and anxiety, and frequent misclassification [8-10]. Beyond its clinical impact, migraine exerts substantial social and economic consequences, including reduced workplace productivity, academic disruption, and compromised social functioning [11].

Despite this high burden, region-specific data, especially among young adults, remain limited, and the condition is inadequately characterized in terms of prevalence, associated factors, and impact.

Available evidence indicates a substantial burden of migraine at the global, national, and regional levels, with a particularly significant impact on young adults in Kerala. Nevertheless, gaps persist in region-specific epidemiological data, especially among college-going populations. Addressing these gaps is crucial for early recognition, timely management, and the development of targeted public health interventions aimed at reducing the personal, social, and economic consequences of migraine. Therefore, the present study was done to estimate the prevalence of migraine among students in selected colleges of Ernakulam district and to identify the factors associated with migraine in this population.

## Materials and methods

This cross-sectional study was conducted over a two-week period during March 2025 among students from selected colleges in Ernakulam district, Kerala. The study population comprised students aged ≥18 years enrolled in one medical, one engineering, and one arts and sciences college. Students aged <18 years were excluded from the study. Students with a prior clinical diagnosis of migraine were not excluded, as the objective was to estimate the overall prevalence of migraine in the study population using a standardized screening tool. Colleges were selected based on feasibility and willingness to participate, and students were approached through the institutional dissemination of the online survey link via official communication channels.

The sample size was calculated based on a migraine prevalence of 30% reported by Raju and Geetha [12]. Using the formula n = Z²pq/d², with Z = 1.96 corresponding to a 95% confidence level, p = 30, q = 70, and d = 5% as the allowable error, the minimum required sample size was estimated to be 336.

Data were collected using a semi-structured, self-administered questionnaire distributed through Google Forms (Google, Inc., Mountain View, CA). To prevent duplicate entries, the Google Forms settings were configured to allow only one response per email account. Mandatory response options were enabled for all key variables, thereby ensuring the completeness of responses before submission.

The questionnaire included sociodemographic details, morbidity history, perceived stress assessment using the Perceived Stress Scale-10 (PSS-10) [13], and migraine screening using the ID-Migraine questionnaire [14], a validated three-item screening tool in which the presence of two or more positive responses indicates probable migraine. The PSS-10 and ID-Migraine questionnaires were selected due to their widespread use and prior validation in diverse populations. Internal consistency was not reassessed in the present study. Formal permission to reproduce the ID-Migraine and PSS-10 was obtained via RightsLink, and the questionnaires have been given in the Appendices.

Ethical approval was obtained from the Ethics Committee of Amrita School of Medicine (approval number: ECASM-AIMS-2024-154). Electronic informed consent was obtained from all participants prior to data collection.

Data were entered into Microsoft Excel (Microsoft Corp., Redmond, WA) and analyzed using Jamovi version 2.6.2 (retrieved from https://www.jamovi.org). Categorical variables are presented as frequencies and percentages, and continuous variables as mean ± standard deviation. Associations between migraine status (ID-Migraine: yes/no) and potential risk factors were examined using the chi-square test. Variables with a p-value of <0.20 in univariate analysis were included in the multivariable logistic regression model to avoid the premature exclusion of potentially important confounders, in accordance with standard epidemiological modeling practices. Categorical variables were entered using dummy coding with clearly defined reference categories. Adjusted odds ratios (aORs) with 95% confidence intervals (CIs) were reported. Model diagnostics included the assessment of goodness of fit using the Hosmer-Lemeshow test. Multicollinearity was assessed using the variance inflation factor (VIF), where a VIF of >5 was considered indicative of multicollinearity. A p-value of <0.05 was considered statistically significant.

## Results

A total of 491 students participated in the study, with a mean age of 20.0 ± 1.44 years. The majority of the participants were aged ≤20 years (n = 338, 68.8%), and women constituted slightly more than half of the sample (n = 256, 52.1%). Nearly half of the students were enrolled in arts courses (n = 242, 49.3%), followed by engineering (n = 196, 39.9%) and medical courses (n = 53, 10.8%). Most participants were day scholars (n = 298, 60.7%) (Table 1).

**Table 1:** Sociodemographic characteristics, lifestyle factors, and self-reported morbidities of the study participants (n = 491) Data are presented as frequency and percentage (n {%}). Age is summarized as mean ± standard deviation *Others: psychiatric illnesses, thyroid disorder, and hypertension

Variables	Category	Frequency n (%)
Age (in years)	≤20	338 (68.8)
	>20	153 (31.2)
Gender	Male	235 (47.9)
	Female	256 (52.1)
Course	Arts	242 (49.3)
	Engineering	196 (39.9)
	Medicine	53 (10.8)
Accommodation	Hostel	193 (39.3)
	Day scholar	298 (60.7)
Sleep (hours/day)	Adequate (≥8)	84 (17.1)
	Inadequate (<8)	407 (82.9)
Physical activity (minutes/week)	Adequate (≥150)	388 (79.0)
	Inadequate (<150)	103 (21.0)
Meals skipped per week	Frequently	91 (18.6)
	Rarely	400 (81.5)
Screen time per day (hours/day)	≤2 hours	28 (5.7)
	>2-6 hours	303 (61.7)
	>6 hours	160 (32.6)
Self-reported morbidities	Allergy	17 (3.5)
	PCOS	8 (1.6)
	Diabetes	1 (0.2)
	Epilepsy	2 (0.4)
	Others*	27 (5.5)
Smoking	Yes	17 (3.5)
	No	474 (96.5)
Alcohol use	Yes	49 (10.0)
	No	442 (90.0)
Substance abuse	Yes	7 (1.4)
	No	484 (98.6)

The assessment of lifestyle factors revealed that inadequate sleep (<8 hours/day) was highly prevalent, reported by 82.9% of students (n = 407). Despite this, a large proportion of participants reported engaging in adequate physical activity (>150 minutes/week) (n = 388, 79.0%). Frequent skipping of meals was reported by 18.6% of the participants (n = 91). Screen exposure was substantial, with nearly one-third of students reporting screen time exceeding six hours per day (n = 160, 32.6%) (Table 1).

Self-reported morbidities were relatively infrequent and included allergies (n = 17, 3.5%), polycystic ovarian syndrome among female students (n = 8, 1.6%), diabetes (n = 1, 0.2%), epilepsy (n = 2, 0.4%), and other conditions such as psychiatric or thyroid disorders (n = 27, 5.5%). Risk-related lifestyle habits were uncommon, with smoking reported by 3.5% of the students (n = 17), alcohol consumption by 10.0% (n = 49), and substance use by 1.4% (n = 7) (Table 1).

Based on the PSS-10, nearly two-thirds of the participants had moderate perceived stress (63.3%; n = 311; 95% CI: 59.0-67.6), while 21.8% experienced high stress levels (n = 107; 95% CI: 18.2-25.5). Only 14.9% of the students reported low perceived stress (n = 73; 95% CI: 11.8-18.1) (Table 2).

**Table 2:** Distribution of the study participants based on PSS-10 (n = 491) Data are presented as frequency and percentage (n {%}). PSS-10 categories were classified as low (0-13), moderate (14-26), and high (27-40) PSS-10: Perceived Stress Scale-10

PSS-10 Category	Frequency, n (%)
Low (0-13)	73 (14.9)
Moderate (14-26)	311 (63.3)
High (27-40)	107 (21.8)

**Figure 1:**
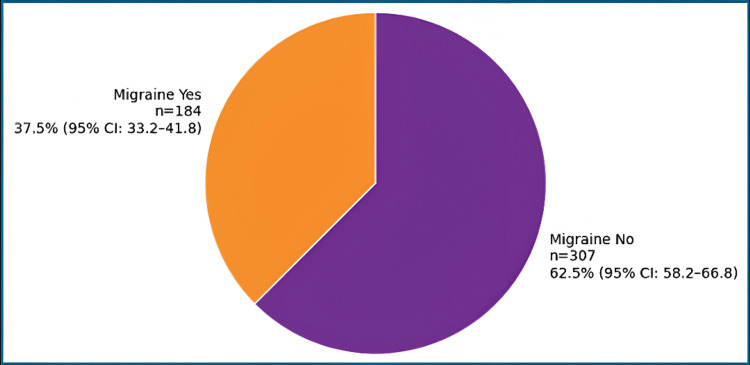
Representation of the prevalence of probable migraine among the study participants using the ID-Migraine questionnaire (n = 491) Data are presented as frequency (n), percentage (%), and 95% confidence interval (CI)

The overall prevalence of probable migraine, as identified using the ID-Migraine questionnaire, was 37.5% (n = 184, 95% CI: 33.2-41.8) (Figure 1).

On univariate analysis, significant associations were observed with inadequate sleep, lower levels of physical activity, the frequent skipping of meals, higher screen time, and higher perceived stress levels. No statistically significant associations were found between migraine and age group, the course of study, the type of accommodation, or substance-related behaviors such as smoking, alcohol consumption, and substance abuse (Table 3).

**Table 3:** Univariate analysis of factors associated with migraine (n = 491) Data are presented as frequency and percentage (n {%}). Crude odds ratios (cOR) with 95% confidence intervals (CIs) are shown. Associations were tested using the chi-square (χ²) test. A p-value of <0.05 was considered statistically significant *Significant p-values PSS: Perceived Stress Scale

Variables	Category	Migraine (Yes), n (%)	Migraine (No), n (%)	Crude OR (95% CI)	χ²	P-value
Age (years)	≤20	127 (37.6)	211 (62.4)	1 (reference)	0.005	0.946
	>20	57 (37.3)	96 (62.7)	0.99 (0.69-1.41)		
Gender	Male	65 (27.7)	170 (72.3)	1 (reference)	16.21	<0.001*
	Female	119 (46.5)	137 (53.5)	2.27 (1.60-3.23)		
Accommodation	Day scholar	108 (36.2)	190 (63.8)	1 (reference)	0.49	0.483
	Hostel	76 (39.4)	117 (60.6)	1.14 (0.80-1.63)		
Course	Arts	93 (38.4)	149 (61.6)	1 (reference)	0.89	0.64
	Engineering	69 (35.2)	127 (64.8)	0.87 (0.60-1.26)		
	Medicine	22 (41.5)	31 (58.5)	1.14 (0.62-2.10)		
Sleep (hours/day)	Adequate (≥8)	19 (22.6)	65 (77.4)	1 (reference)	9.63	0.002*
	Inadequate (<8)	165 (40.5)	242 (59.5)	2.34 (1.35-4.06)		
Physical activity (minute/week)	Adequate (≥150)	136 (35.1)	252 (64.9)	1 (reference)	4.67	0.031*
	Inadequate (<150)	48 (46.6)	55 (53.4)	1.62 (1.05-2.50)		
Meals skipped/week	Rarely	140 (35.0)	260 (65.0)	1 (reference)	5.63	0.018*
	Frequently	44 (48.4)	47 (51.6)	1.74 (1.10-2.75)		
Screen time (hours/day)	>6 hours	73 (45.6)	87 (54.4)	1 (reference)	6.75	0.034*
	≤2 hours	10 (35.7)	18 (64.3)	0.66 (0.29-1.49)		
	2-6 hours	101 (33.3)	202 (66.7)	0.60 (0.41-0.88)		
Smoking	No	175 (36.9)	299 (63.1)	1 (reference)	1.79	0.18
	Yes	9 (52.9)	8 (47.1)	1.92 (0.72-5.10)		
Alcohol use	No	163 (36.9)	279 (63.1)	1 (reference)	0.67	0.412
	Yes	21 (42.9)	28 (57.1)	1.28 (0.73-2.25)		
Substance abuse	No	181 (37.4)	303 (62.6)	1 (reference)	0.09	0.767
	Yes	3 (42.9)	4 (57.1)	1.26 (0.28-5.70)		
Stress level (PSS)	Low (0-13)	8 (11.0)	65 (89.0)	1 (reference)	45.92	<0.001*
	Moderate (14-26)	109 (35.0)	202 (65.0)	4.39 (2.00-9.70)		
	High (27-40)	67 (62.6)	40 (37.4)	13.60 (5.80-31.80)		

In multivariable logistic regression analysis, perceived stress, gender, sleep duration, and screen time were independently associated with probable migraine (Table 4). Students with high perceived stress had more than 10-fold higher odds of migraine compared to those with low stress (aOR: 10.23; 95% CI: 4.38-23.89), while moderate stress was associated with nearly fourfold higher odds (aOR: 3.74; 95% CI: 1.73-8.36). Female students were more than twice as likely to report migraine compared to men (aOR: 2.21; 95% CI: 1.46-3.34). Inadequate sleep (<8 hours/day) was independently associated with migraine (aOR: 2.10; 95% CI: 1.17-3.75). With respect to screen exposure, students reporting prolonged screen time (>6 hours/day) had significantly higher odds of migraine (aOR: 1.75; 95% CI: 1.13-2.70), whereas those reporting screen time of ≤2 hours/day had 1.05 times higher odds, but this was not found to be statistically significant (95% CI: 0.44-2.52). The multivariable logistic regression model demonstrated an adequate fit to the data (Hosmer-Lemeshow p = 0.289, Nagelkerke's R-squared {NR^2^} = 0.204). All variables included in the model had VIF values of <5, indicating no evidence of multicollinearity (Table 4).

**Table 4:** Multivariable logistic regression analysis of factors associated with migraine (n = 491) Results are presented as adjusted odds ratios (aOR) with 95% confidence intervals (CIs). A p-value of <0.05 was considered statistically significant *Significant p-values PSS: Perceived Stress Scale

Variables	Category	Migraine (Yes), n (%)	Migraine (No), n (%)	aOR (95% CI)	P-value
Sex	Male	65 (27.7)	170 (72.3)	1	<0.001*
	Female	119 (46.5)	137 (53.5)	2.21 (1.46-3.34)	
Sleep (hours/day)	Adequate (≥8)	19 (22.6)	65 (77.4)	1	0.012*
	Inadequate (<8)	165 (40.5)	242 (59.5)	2.10 (1.17-3.75)	
Screen time	≤2 hours	10 (35.7)	18 (64.3)	1.05 (0.44-2.52)	0.899
	3-6 hours	101 (33.3)	202 (66.7)	1	-
	>6 hours	73 (45.6)	87 (54.4)	1.75 (1.13-2.70)	0.011*
Stress level (PSS)	Low (0-13)	8 (11.0)	65 (89.0)	1	-
	Moderate (14-26)	67 (62.6)	40 (37.4)	3.74 (1.73-8.36)	0.001*
	High (27-40)	109 (35.0)	202 (65.0)	10.23 (4.38-23.89)	<0.001*

## Discussion

This study evaluated the prevalence of probable migraine and its associated factors among college students in Ernakulam district, Kerala. The sociodemographic profile of the study population is comparable to other university-based studies conducted in South Asia, with a young mean age and a slight female predominance. Similar age distributions have been reported among university students in Bangladesh and South India, reflecting the vulnerability of young adults to migraine during academically demanding years [12,15]. The higher representation of female students in the present study is noteworthy, as migraine is consistently reported to be more common among women across different populations [2,8].

Perceived stress emerged as an important characteristic of the study population. Based on the PSS-10, nearly two-thirds of the participants reported moderate stress, while more than one-fifth experienced high perceived stress. This finding aligns with evidence suggesting that university students are exposed to considerable academic and psychosocial stressors, which may predispose them to stress-related neurological disorders such as migraine [5,8]. Stress is known to influence migraine pathophysiology through neuroendocrine mechanisms involving the hypothalamic-pituitary-adrenal axis and altered pain modulation pathways [1,5].

The overall prevalence of probable migraine in this study was 37.5% (n = 184; 95% CI: 33.2-41.8), which is higher than that reported in several international studies. For example, Rafi et al. [15] reported a prevalence of 21.4% among university students in Bangladesh, while a systematic review and meta-analysis from South America documented a pooled prevalence of 19% among university students [16]. Indian studies among medical students have reported prevalence estimates ranging from 28% to 30% [12]. The comparatively higher prevalence observed in the present study may reflect differences in lifestyle patterns, academic stress, urban environmental exposures, or the use of a sensitive screening tool such as ID-Migraine, which has been validated for epidemiological studies [14].

On univariate analysis, migraine was significantly associated with gender, sleep, physical activity, meal skipping, screen time, and perceived stress. These findings are consistent with previous literature identifying lifestyle and behavioral factors as important migraine triggers in student populations [11,15,17]. Sleep deprivation, in particular, has been strongly linked to migraine; a Chinese study among college students demonstrated a significant association between poor sleep quality and migraine [18]. Similarly, excessive screen exposure has been recognized as a modern risk factor, with Montagni et al. reporting higher migraine prevalence among students with prolonged daily screen time [19].

Multivariable logistic regression analysis further demonstrated that perceived stress, female gender, inadequate sleep, and screen time were independently associated with probable migraine. Students with high perceived stress had markedly higher odds of migraine compared to those with low stress, underscoring the central role of psychological stress in migraine occurrence. The observed female predominance is consistent with established epidemiological evidence and is likely related to hormonal influences and differential stress responses [2,8].

The strengths of this study include the use of well-validated screening instruments, namely, the PSS-10 and the ID-Migraine questionnaire, both of which have been widely employed in epidemiological research. The inclusion of students from diverse academic disciplines, including medicine, engineering, and arts and sciences, allowed the representation of varied academic environments and lifestyle contexts, thereby enhancing the heterogeneity and comparability of the study population with global college student cohorts. Furthermore, the observed associations are consistent with findings from previous studies, reinforcing the relevance of modifiable lifestyle factors, such as inadequate sleep and excessive screen exposure, in the burden of migraine among students.

The study was conducted in selected urban colleges using a convenience sampling approach, which may limit the generalizability of the findings to students in rural or semi-urban settings. Migraine status was assessed using a self-reported screening questionnaire rather than clinical confirmation, which may have introduced misclassification bias. In addition, the internal consistency of the PSS-10 and ID-Migraine questionnaires was not reassessed in the present study. The screen time category of ≤2 hours/day included a relatively small number of participants, which may have limited the precision and stability of the corresponding estimates and should therefore be interpreted with caution. Furthermore, certain potential confounders, such as family history of migraine, caffeine intake, and psychiatric comorbidities, were not assessed. Although the inclusion of students from multiple colleges introduces heterogeneity, clustering effects were not examined, as the study was not designed or powered for multilevel analysis. Despite these limitations, the study highlights migraine as a significant and underrecognized public health concern among college students in Kerala.

## Conclusions

This study highlights that probable migraine is a common health problem among college students in Ernakulam district, with a higher prevalence observed among women and among students reporting inadequate sleep, prolonged screen exposure, and elevated perceived stress. These factors were found to be significantly associated with probable migraine in this population.

From a public health and clinical perspective, the findings underscore the importance of incorporating migraine screening, stress management strategies, sleep hygiene education, and guidance on healthy screen-use practices within student health services and primary care settings. The early identification of students with probable migraine and associated lifestyle factors may help reduce academic disruption and improve the quality of life. Further large-scale studies, particularly those including rural and semi-urban populations and employing longitudinal designs, are warranted to better understand the temporal relationships and broader determinants of migraine among young adults.

## Figures and Tables

**Figure 2:**
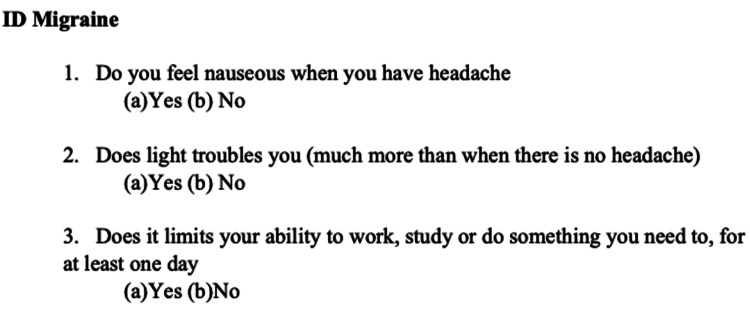
ID-Migraine screening tool The ID-Migraine questionnaire is a validated three-item screening instrument developed to identify individuals with probable migraine in community and primary care settings. ©2003 Wolters Kluwer Health, Inc. Reproduced with permission. ID-Migraine is a trademark of the American Academy of Neurology

**Figure 3:**
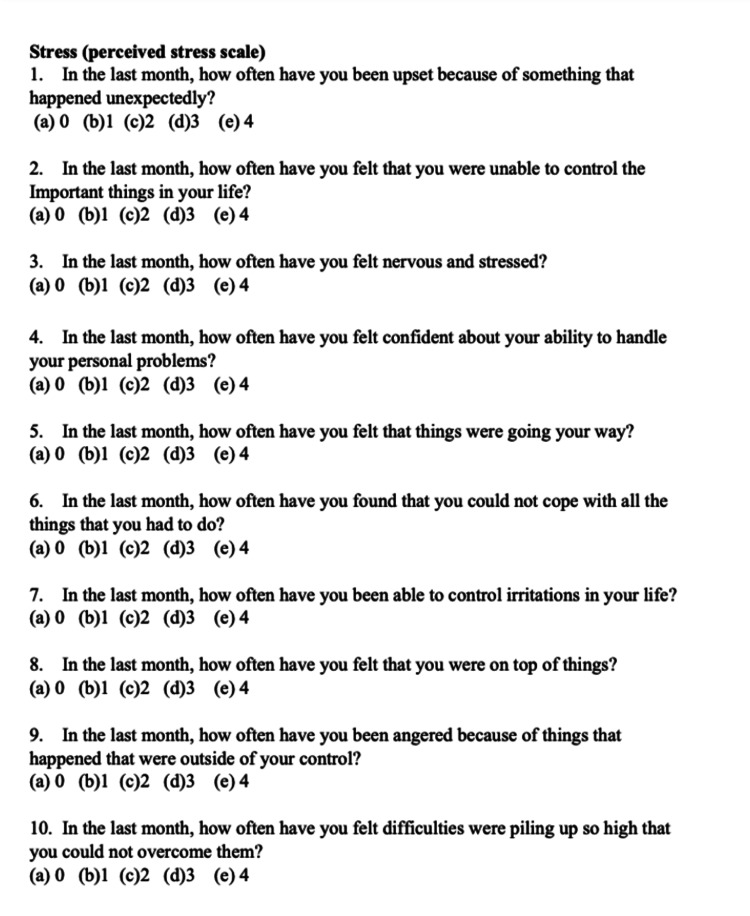
Perceived Stress Scale-10 (PSS-10) The Perceived Stress Scale-10 (PSS-10) is a widely used, validated self-administered questionnaire designed to assess the degree to which individuals perceive situations in their lives as stressful over the preceding month. ©Cohen S, Kamarck T, and Mermelstein R. Perceived Stress Scale. Reproduced with permission

## Appendices

Figure 2 shows the ID-Migraine questionnaire.

 Figure 3 shows the Perceived Stress Scale-10.
